# Pancreatic Juice as a Therapeutical Agent

**Published:** 1873-06

**Authors:** 


### Miscellaneous.

### Pancreatic Juice as a Therapeutical Agent.

The increasing employment of the pancreatic juice in pharmacy renders any process for its preparation valuable, specially when such process is within the reach of practitioners in even the most remote countıy districts. Dr. Merkel, of Nuremberg, has given the following directions for preparing the pancreatic fluid : The pancreas of a bullock is finely minced and rubbed up with 250 grammes (about 8 ounces) of glycerine. When used as a nutritive enema, one-third of this is thoroughly mixed with from 120 to 150 grammes (a gramme is 15.5 grs.) of finely minced meat, and immediately in jected into the rectum. This said to be readily digested by the intestines. If the mixture is allowed to stand too long before using, the meat swells up and renders the injecting difficult.

For feeding by the mouth in the treatment of the wasting diseases, both of children and of adults, we would suggest that a very satisfactory pancreatic fluid can be prepared as follows : The pancreas of either the bullock or pig may be used.

A pound of fresh pancreas is finely minced, and an equal quantity of glycerine added at about 120° Fahrenheit. The mixture is put in a vessel, when the pancreas can be crushed and intimately mixed with the glycerine, and kept at this temperature in a water-bath for twenty-four hours, being occasionally stirred. The glycerine is then carefully drained off, as completely as possible, and the pancreatic magma is carefully washed with about six or eight ounces of water, so as to extract the remainder of the glycerine. This is also carefully drawn off and filtered, and evaporated to one half, and then mixed with the glycerine.

This makes a perfect and very agreeable emulsion, with cod-liver oil or any other form of fat. Cod-liver oil mixed with this, in the proportion of one part to ten, gives a fine and palatable emulsion, very easy of digestion.

Cod-liver oil, it is said, may also be successful emulsified, by adding to it crushed bits of pancreas, and keeping it at a temperature of 140° Fahrenheit for six hours; thus emulsified, it rapidly passes into the circulation from the stomach.

It is well known that nearly all the cases of wasting disease die from pure inanition and complete defection and incapacity of appropriation and nutrition. Very many of these cases will recover if we can but present material to the assimilating organs readily prepared for absorption. By this means, new impulses of force are acquired, by the entrances of those materials into the chemico-vital action common to organic bodies.

By mixing one or two teaspoonfuls of this glycerine extract with a half pint of milk, thickened with a mixture of three parts wheat flour, and one part pure malt flour, we have a pure and perfectly assimilable diet for all such cases, on which they thrive remarkably.

The cod-liver oil emulsified by this pancreatic extract forms the basis of a most successful treatment in all wasting diseases, under which eighty per cent. will recover.—*Medical Archives.*—*Druggists' Circular.*

